# Targeted Cancer Therapy with a Novel Anti-CD37 Beta-Particle Emitting Radioimmunoconjugate for Treatment of Non-Hodgkin Lymphoma

**DOI:** 10.1371/journal.pone.0128816

**Published:** 2015-06-11

**Authors:** Ada H. V. Repetto-Llamazares, Roy H. Larsen, Sebastian Patzke, Karianne G. Fleten, David Didierlaurent, Alexandre Pichard, Jean Pierre Pouget, Jostein Dahle

**Affiliations:** 1 Nordic Nanovector ASA, Kjelsåsveien 168, 0884, Oslo, Norway; 2 Department of Radiation Biology, Institute for Cancer Research, Oslo University Hospital, Montebello, 0310, Oslo, Norway; 3 Sciencons AS, Kjelsåsveien 168, 0884, Oslo, Norway; 4 Department of Tumor Biology, Institute for Cancer Research, Oslo University Hospital, Montebello, 0310, Oslo, Norway; 5 UMR 1037 INSERM/UPS, Centre de Recherche en Cancérologie de Toulouse, Toulouse, F-31062, France; 6 Institut de Recherche en Cancérologie de Montpellier, Institut National de la Santé et de la Recherche Médicale, U896, Université Montpellier, Montpellier, France; Queen's University Belfast, UNITED KINGDOM

## Abstract

^177^Lu-DOTA-HH1 (^177^Lu-HH1) is a novel anti-CD37 radioimmunoconjugate developed to treat non-Hodgkin lymphoma. Mice with subcutaneous Ramos xenografts were treated with different activities of ^177^Lu-HH1, ^177^Lu-DOTA-rituximab (^177^Lu-rituximab) and non-specific ^177^Lu-DOTA-IgG_1_ (^177^Lu-IgG_1_) and therapeutic effect and toxicity of the treatment were monitored. Significant tumor growth delay and increased survival of mice were observed in mice treated with 530 MBq/kg ^177^Lu-HH1 as compared with mice treated with similar activities of ^177^Lu-rituximab or non-specific ^177^Lu-IgG1, 0.9% NaCl or unlabeled HH1. All mice injected with 530 MBq/kg of ^177^Lu-HH1 tolerated the treatment well. In contrast, 6 out of 10 mice treated with 530 MBq/kg ^177^Lu-rituximab experienced severe radiation toxicity. The retention of ^177^Lu-rituximab in organs of the mononuclear phagocyte system was longer than for ^177^Lu-HH1, which explains the higher toxicity observed in mice treated with ^177^Lu-rituximab. *In vitro* internalization studies showed that ^177^Lu-HH1 internalizes faster and to a higher extent than ^177^Lu-rituximab which might be the reason for the better therapeutic effect of ^177^Lu-HH1.

## Introduction

Despite the promise of therapy using the naked monoclonal antibody (mAb) rituximab, a substantial number of the patients treated with conventional doses of rituximab alone or in combination with chemotherapy do not obtain complete response and may eventually relapse [1]. Alternative treatments have been anti-CD20 mAbs conjugated to ^131^I (tositumomab) or ^90^Y (ibritumomab-tiuxetan). Treatment with conventional activities of the radiolabeled mAbs has produced higher overall response and complete remission rates compared with naked mAbs [2–5]. Considering that radioimmunotherapy (RIT) is mostly used after patients have been treated with several rounds of rituximab and that the two approved radioimmunoconjugates (RICs) for clinical use, ^90^Y-ibritumomab-tiuxetan (Zevalin) and ^131^I-tositumomab (Bexxar), target the same CD20 antigen as rituximab, it is desirable to design a new RIC that will target a different antigen than CD20. The CD37 antigen is abundantly expressed in B-cells, but is absent on plasma cells and normal stem cells [6–8]. Therefore, CD37 seems to be an appropriate therapeutic target in patients with relapsed B-cell derived malignancies, such as B-cell CLL, hairy-cell leukemia (HCL) and B-cell NHL.

RIT with CD37 as target has previously been explored using a ^131^I-labeled murine monoclonal antibody (MB-1) both in a mouse model and in patients [9–14]. A higher degree of internalization and degradation of ^131^I-labeled RIC was found for CD37 than for CD20 [14]. Despite promising clinical responses observed in these clinical studies for the anti-CD37 antibody, further development of RIT focused on CD20 as the target antigen and no subsequent efforts have been made to develop RIT with anti-CD37-based RICs. A limited number of other CD37-directed antibody based immunotherapies have, however, been evaluated in patients. The small modular immunopharmaceutical protein Otlertuzumab has advanced into clinical testing [15] and recently reported on phase II data in combination with bendamustine [16]. In addition, the Fc-engineered antibody CD37.1 (BI836826) [17] has recently entered phase I [18]. Furthermore, two antibody-drug conjugates (ADCs) have been developed that covalently link cytotoxic agents to CD37-targeting antibodies to enhance their antitumor potency: IMGN529 [19] and AGS-67E [20]. ADCs are designed to give specific delivery of cytotoxic compounds to cells expressing the target antigen, through ADC binding, internalization, and intracellular payload release. Clinical data have demonstrated the potential of ADCs for cancer therapy of CD30 and HER2 positive tumors [21,22]. All these CD37 targeting drugs had shown promising results, which further validates CD37 as a target for treatment of NHL and CLL. An advantage with RIT compared with naked mAbs and ADCs is the range of the emitted radiation, which gives a cross-fire effect so that tumor cells with less antigens or non-accessible tumor cells also get hit by the cytotoxic radiation. It remains to be seen if the mechanism of action of RIT is better than that of ADCs.

The potency of RIT against the internalizing antigen CD37 might have been underestimated by the use of the radionuclide ^131^I, which tends to be cleaved off from the antibody and excreted from the cells upon internalization and catabolism when used as “non-residualizing” tyrosine-incorporated radiolabel, as was done in the early studies with ^131^I-MB-1 [23]. “Residualizing” radiolabels, on the other hand, are trapped in the cells after metabolism of the RIC. In an effort to re-evaluate and improve RIT against CD37 we have developed a new RIC (Betalutin) based on the “residualizing” radiolabel ^177^Lu linked to the anti-CD37 antibody HH1 [24]. Treatment with 100 MBq/kg ^177^Lu-HH1 resulted in a threefold increase in the survival of SCID mice that were intravenously injected with Daudi lymphoma cells compared to untreated control mice [7]. SCID mice are not able to repair DNA double strand breaks [25], limiting the amount of radioactivity that can be administered, while nude mice can tolerate higher doses of radiation. A subcutaneous tumor xenograft in nude mice is a more relevant model for the bulky type of disease that is often found in NHL patients than the intravenous model in SCID mice. Therefore, the therapeutic and toxicity effect of ^177^Lu-HH1 was evaluated in nude mice with subcutaneous Ramos xenografts in the present paper.

## Materials and Methods

### Tumor cells

Ramos lymphoma cells (LGC Standards, Boras, Sweden) expressing the CD20 and CD37 receptors were grown in RPMI 1640 medium supplemented with Glutamax (Gibco, Paisley, UK), 10% heat-inactivated FCS (Gibco) and 1% penicillin-streptomycin (Gibco) in a humid atmosphere with 95% air/5% CO_2_.

### Radiolabeling of antibodies

The antibodies HH1 (anti-CD37, Nordic Nanovector ASA, Oslo, Norway), Rituximab (anti-CD20, Roche, Pharma Schweiz, Basel, Switzerland) and the murine non-specific isotype control antibody (IgG_1_) (MAB002, R&D Systems Inc., Minneapolis, USA) were labeled with the chelator p-SCN-Bn-DOTA (DOTA, Macrocyclics, TX, USA) and subsequently labeled with ^177^Lu (ITG, Garching, Germany) as previously described [26]. Labeling with ^125^I (Hartmann Analytic, Braunschweig, Germany) was performed using iodogen tubes (Pierce, Rockford, IL, USA) as described previously [23].

The immunoreactivity (IRF) of all the tumor specific RICs was verified using a modified Lindmo method [27] with one cell concentration of 75 million cells/ml. The IRF of all specific RICs was between 57% and 76%.

### Number of cell surface receptors

Scatchard analyses were performed using ^177^Lu-HH1 or ^125^I-rituximab. A concentration of 10 million cells/ml of Ramos lymphoma cells were incubated for 1 hour with increasing amounts of radioimmunoconjugates (between 0 and 6.25 nM). Cells previously blocked with unlabeled antibody were used to account for non-specific binding. Each sample was measured in duplicates and the results were averaged. After incubation with the RIC, the activity in each sample was measured using a gamma counter (Cobra gamma; Packard Instrument Co, Meriden, CT, USA) and cells were washed two times using DPBS (Gibco, Paisley, UK) supplemented with 0.5%wt Bovine Serum Albumin (BDH Prolabo, VWR, Lutterworth, Leicestershire, UK), after which the activity bound to the cells was counted in the gamma counter. The equilibrium dissociation constant (K_d_) and maximum average density of antigens (B_max_) was calculated from the fitting of the experimental data. Three experiments were performed using ^177^Lu-HH1 and 2 experiments using ^125^I-rituximab. The results from all experiments for each RIC were averaged to obtain an average value of K_d_ and B_max_ for each RIC.

### Internalization experiments

HH1-DOTA was labeled with Alexa Fluor 488 and rituximab with Alexa Fluor 647 (Molecular Probes, Invitrogen, Paisley, UK). One million Ramos cells per ml in growth medium were incubated with 10 μg/ml of HH1 or 20 μg/ml of rituximab for either 19 hours at 37°C or 1 h at around 4°C. Hoechst 33342 (Sigma-Aldrich, St.Louis, MO, US) was added to the cells to a concentration of 2 μg/ml 1 hour before imaging. Cells were imaged with an AxioImager Z1 ApoTome microscope system (Carl Zeiss, Jena, DE) using a PlanApo 63×/N.A.1.4 DIC lens, appropriate optical filters, an AxioCam MRm camera and Axiovision 4.8.2 (Carl Zeiss). Z-sections were obtained for every 1 μm across the entire cell volume. Images are presented as overlays of fluorescence images of central z-section and DIC image. 20 to 40 cells were scanned for each treatment.

### Animals and xenografts

Institutionally bred female Athymic nude FOX1^nu^ mice were used. Age and weight of the mice are given in Table 1. The animals were maintained under pathogen-free conditions with a 12 hours lighting cycle at a room temperature of 23°C and air relative humidity of 55% in plastic cages. Food and water were supplied *ad libitum* and bedding was changed regularly. Mice were anesthetized with subcutaneous injections of 70–100 μl Tiletamin-Zolazepam mix (Virbac, Carros Cedex, France) diluted 1:5 with sterile water before implantation of pieces of Ramos lymphoma xenograft tissue from carrier mice, (diameter 1.5–2 mm) in the flanks. All procedures and experiments involving animals in this study were approved by The Norwegian Animal Research Authority (NARA). The Department of Comparative Medicine institutional veterinarian has established the rules for feeding, monitoring, handling, and sacrifice of animals in compliance with regulations set by the Ministry of Agriculture of Norway and “The European Convention for the Protection of Vertebrate Animals used for Experimental and other Scientific Purposes”. The institutional veterinarian has delegated authority from the Norwegian Animal Research Authority (NARA). The laboratory animal facilities are subject to a routine health-monitoring program and tested for infectious organisms according to a modification of Federation of European Laboratory Animal Science Associations (FELASA) recommendations.

**Table 1:** Overview of therapy and toxicity experiments in nude mice with Ramos xenografts.

Experiment	Treatments	mice/group	Mice age^a^ (weeks)	Mice weights^b^ (g)	Tumor volumes^b^, (mm^3^)
1	530 MBq/kg ^177^Lu-HH1, 530 MBq/kg ^177^Lu-rituximab, 530 MBq/kg ^177^Lu-IgG1, 15 μg/kg HH1, 0.9% NaCl	9–10	6–8	21.1–29.0 (25 ± 2)	20–780 (139 ± 134)
2	410 MBq/kg ^177^Lu-HH1, 300 MBq/kg ^177^Lu-rituximab, 0.9% NaCl.	5–6	6–8	21.0–29.7 (25 ± 2)	22–760 (342 ± 221)

^a^At implantation of xenografts.

^b^At treatment injection Min-Max (Average ± SD).

### Therapy and toxicity experiments

Animals bearing growing tumors with diameters between 4 and 12 mm were randomized in cages according to treatment group and were administered intravenously with 100 μl of injection solutions adjusted for individual mouse weight. Two preliminary experiments were performed with injection of activities between 50 and 1000 MBq/kg ^177^Lu-HH1 (S1 File). These experiments were used to decide the experimental parameters chosen for the two experiments presented in this paper (Table 1) which included injected activities of 410 and 530 MBq/kg ^177^Lu-HH1, 300 and 530 MBq/kg ^177^Lu-rituximab, 530 MBq/kg ^177^Lu-IgG_1_ and 15 μg/kg HH1 and 0.9% NaCl as control groups.

The mice were weighed and clinically observed two to three times a week for up to 109 days. In addition the mice were monitored daily for any sign of illness or discomfort. Activity level, skin condition (eczema, hemorrhages, etc.) and general health of the mice were observed daily. Mice were euthanized by cervical dislocation under Sevoflurane gas anesthesia if any of the following humane end points were reached: tumor diameter exceeded 20 mm, body weight decreased by 20% from highest body weight or animals otherwise showed symptoms of severe illness and discomfort.

Tumor growth was monitored up to the first tumor volume measurement higher than 2000 mm^3^ in each mouse (bigger tumors usually develop necrotic wounds). Complete remission was defined as no observable tumor. Mice reaching this stage were considered as long term responders.

#### Hematology

Blood samples were taken prior to injection, at death and every 4 to 7 weeks after treatment administration. The blood sampling was performed as described previously [26]. Radiation toxicity was defined as white blood cell counts below 1×10^9^ 1/L, platelet counts below 400×10^9^ 1/L, sudden weight loss—around 5% in the lapse of 2 to 3 day- accompanied by a low activity level, and a general state of sickness. Comparisons between average values of different treatment groups were done using t-tests with a significance level of 0.05.

One mouse treated with 530 MBq/kg ^177^Lu-HH1 was sacrificed 11 days after treatment injection in order to obtain hematology and histology data at this particular time point. Furthermore, to obtain more hematological data 3 mice having tumor diameters between 12 and 15 mm (800 and 1300 mm3) were also included in experiment 2.

#### Histopathology

Mice were necropsied and heart, lungs, liver, stomach, spleen, small and large intestines, kidneys, femur, muscle, skull, brain, tumor, skin, ovaries and lymph nodes were fixed and shipped to Accelera, Milano, Italy for casting, sectioning, staining and pathological examination.

### Biodistribution experiments

Conjugates were administered by tail vein injection of 100 μl solution (0.2 and 0.9 MBq) to each animal. Between 4 and 7 mice were sacrificed by neck dislocation under Sevoflurane gas anesthesia at each time point after cardiac puncture for blood sampling. Tumor sizes at injection of conjugates varied between 120 and 1800 mm^3^. Studies were conducted as described in reference [28]. Comparisons between average values were done using t-tests with a significance level of 0.05.

A pilot *In vivo* SPECT/CT was performed on 2 mice with Ramos subcutaneous xenografts with diameters around 12 mm in their right flank in a nanoSPECT/CT (Bioscan Inc., Washington DC, USA). SPECT/CT acquisitions were performed at 3, 24 and 48 hours after treatment injection in mouse 1 and after 24 hours in mouse 2. Activities measured by SPECT/CT were compared with ex-vivo measurements done at 48 hours after treatment administration. Mice were injected i.v. with 150 μl of 10 MBq of ^177^Lu-HH1 and were anaesthetized with 2% Isofluran for image acquisition.

### Dosimetry

The dose rate and absorbed doses from ^177^Lu-HH1 and ^177^Lu-rituximab in mice were calculated as described in reference [28] for an injection of 1 MBq/mouse (40 MBq/kg) and assuming the mean energy of the β-particles, Auger- and conversion electrons to be 0.1473 MeV [29]. The self-radiation fraction for tumor was estimated to be 0.944 (200 mg tumor [30]).

### Statistical analysis

The survival of the different treatment groups was compared by Kaplan-Meyer survival analysis using SigmaPlot for Windows version 12.0 (Systat Software Inc., California, USA) using time to reach a tumor volume higher than 4 times the initial tumor volume or euthanasia due to radiation toxicity symptoms as the end points. Hematology and biodistribution data were compared using one-way ANOVA. Multiple comparisons were performed using the Holm Sidak method. A significance level of p < 0.05 was used in all tests.

## Results

### Therapy and toxicity experiments

#### Tumor growth and survival

**Fig 1:**
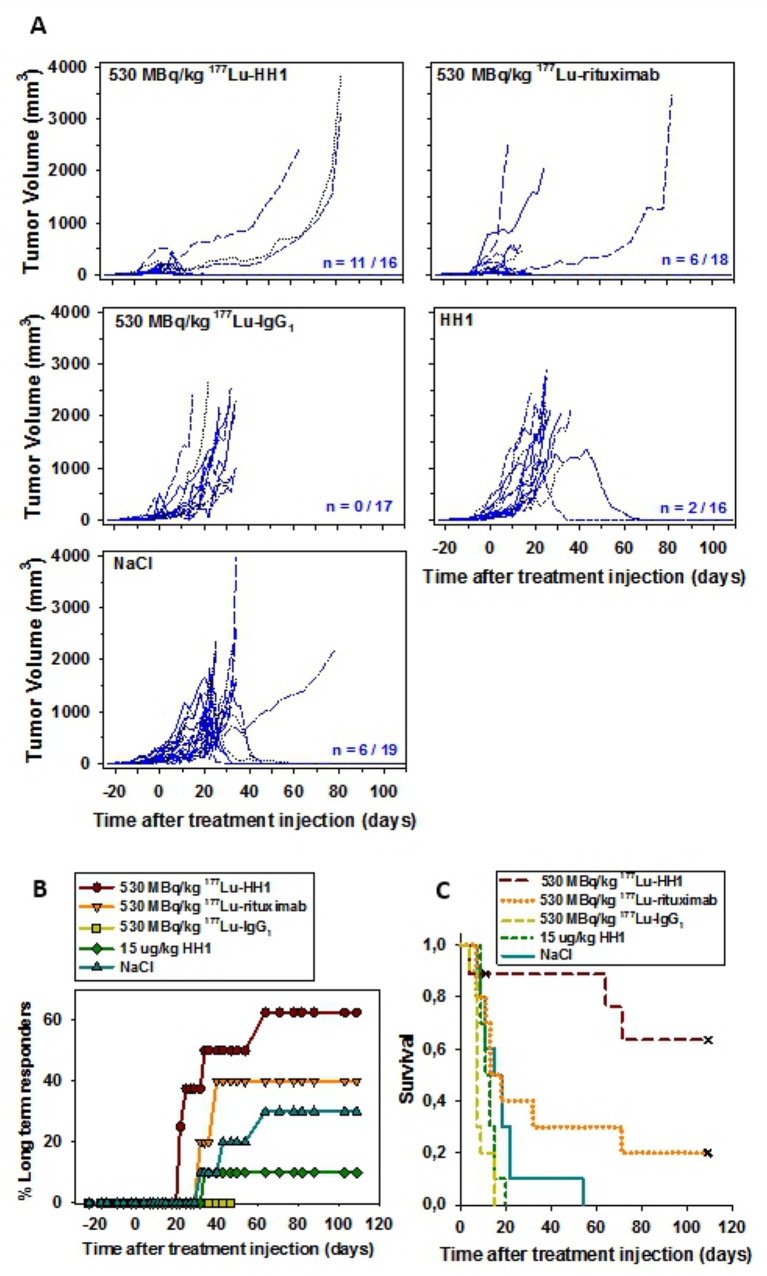
Tumor growth, long term responders and survival of nude mice with Ramos xenografts after treatment with 530 MBq/kg ^177^Lu-HH1, 530 MBq/kg ^177^Lu-rituximab, 530 MBq/kg ^177^Lu-IgG_1_ non-specific isotype control, 15 μg/kg HH1 and 0.9% NaCl. (A) Individual tumor growth (n = Number of tumors in remission at the end of study/total number of initial tumors); (B) Percentage of long term responders (mice euthanized due to radiation toxicity symptoms or for hematology measurements before one of their tumors reached a volume of 2000 mm^3^ were not considered in the analysis); (C) Kaplan-Meier survival curve. End point: tumor volume ≥ 4 times initial tumor volume. Crosses represent censored animals that were euthanized before reaching 4 times the initial tumor volume. N = 9–10. Multiple comparisons were performed using Holm Sidak method. Significance level: p < 0.05.

There was a marked tumor growth delay in mice treated with 530 MBq/kg ^177^Lu-HH1 when compared to mice treated with either ^177^Lu-IgG_1_ or 0.9% NaCl (Fig 1A). Tumor growth was also delayed in some of the mice treated with 530 MBq/kg ^177^Lu-rituximab, but this treatment proved to be toxic: 60% of the mice had to be sacrificed due to symptoms of severe radiation toxicity between 11 and 18 days after injection of treatment (Table 2). None of the mice were sacrificed due to radiation toxicity symptoms in any of the other treatment groups. Tumor growth in the control mice treated with 0.9% NaCl or 15 μg/kg naked HH1 was non-homogeneous, with 30% and 10% complete remission, respectively. On the other hand, tumors in mice treated with 530 MBq/kg ^177^Lu-IgG_1_ grew homogeneously and did not show any natural regressions. The number of long term responders was highest in mice treated with 530 MBq/kg ^177^Lu-HH1 (Fig 1B). Furthermore, treatment with 530 MBq/kg ^177^Lu-HH1 significantly extended the survival of mice compared with ^177^Lu-IgG_1_, 0.9% NaCl and HH1 (p < 0.05) (Fig 1C, Table 2). Survival of mice treated with ^177^Lu-IgG_1_ was significantly shorter than for those treated with 0.9% NaCl (p < 0.05), which was due to the growth homogeneity and the lack of tumor regression observed in mice treated with ^177^Lu-IgG_1_, which might be related to radiation induced immunosuppression. It has been previously shown that growth of xenografts in mice can be enhanced by whole body irradiation (WBI) [31–34] or other immunomodulating agents [32,35–38]. Natural regressions of xenografts in nude mice in control groups have been observed previously [33,35], especially in nude mice with subcutaneous Ramos xenografts [39,40]. RIT can be regarded as a type of WBI treatment, which may therefore result in better take and growth of xenografts in treated mice than in control mice. Thus, the therapeutic efficacy of RIT might be underestimated in mouse models where total body irradiation enhances tumor growth, especially when comparing the therapeutic effect of RICs with agents having low systemic toxicity such as NaCl or unlabeled mAbs. It might be more convenient, therefore, to compare the effect of RICs to corresponding dosages of non-specific, control RICs instead.

**Table 2:** Median survival time of mice and percentage of mice euthanized due to radiation toxicity after treatment with 530 MBq/kg ^177^Lu-HH1, ^177^Lu-rituxmab and ^177^Lu-IgG_1_ non-specific isotype control, HH1 or NaCl.

Experiment	Group	Median survival time ± SE (days)	Radiation Toxicity (%)
1	NaCl	18 ± 3^a^	0
	HH1 15 μg/kg	11 ± 2^a^	0
	530 MBq/kg ^177^Lu-IgG1	7 ± 1^a^	0
	530 MBq/kg ^177^Lu-rituximab	18 ± 4	60
	530 MBq/kg ^177^Lu-HH1	> 109	0
2	NaCl	6 ± 3	0
	300 MBq/kg ^177^Lu-rituximab	17 ± 3	0
	410 MBq/kg ^177^Lu-HH1	> 97	0

^a^Significantly different from ^177^Lu-HH1 (p < 0.05).

**Fig 2:**
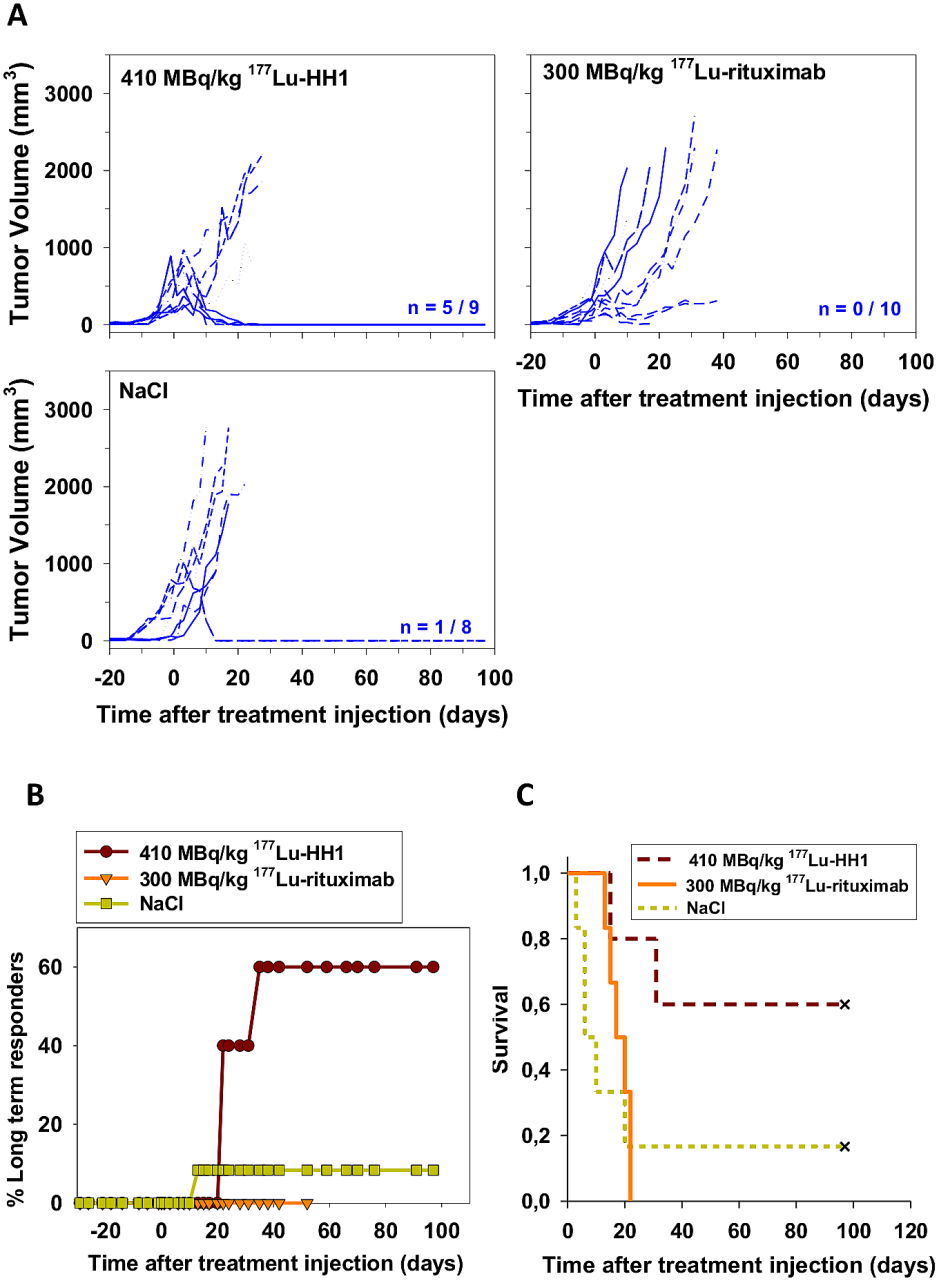
Tumor growth, long term responders and survival of nude mice with Ramos xenografts after treatments with 410 MBq/kg ^177^Lu-HH1, 300 MBq/kg ^177^Lu-rituximab and 0.9% NaCl. (A) Individual tumor growth (n = Number of tumors in remission at the end of study/total number of initial tumors) (B) Percentage of long term responders; (C) Kaplan-Meier survival curve. End point: tumor volume ≥ 4 times initial tumor volume. Crosses represent censored animals that were euthanized before reaching 4 times the initial tumor volume. N = 5–6. Multiple comparisons were performed using Holm Sidak method. Significance level: p < 0.05.

Given the high toxicity observed during the first two weeks in some of the mice treated with ^177^Lu-rituximab, one more experiment was performed using lower activities. Treatments with 410 MBq/kg ^177^Lu-HH1 and 300 MBq/kg ^177^Lu-rituximab (approximately 50% of the LD50 of each RIC) resulted in a slight tumor growth delay (Fig 2A). Mice treated with 410 MBq/kg ^177^Lu-HH1 had the highest number of long term responders while there was no long term responders in mice treated with 300 MBq/kg ^177^Lu-rituximab due to no inhibition of tumor growth (Fig 2B). Median survival was longer in mice treated with 410 MBq/kg ^177^Lu-HH1 (more than 15 times compared to the control group) but the difference was not statistically significant compared with the other treatment groups (p > 0.05) (Fig 2C, Table 2). Median survival of mice treated with 300 MBq/kg ^177^Lu-rituximab was around 3 times longer than for the mice in the control group, while the median survival of mice treated with 530 MBq/kg ^177^Lu-rituximab was similar to the mice in the control group. Even when the amount of long term responders was higher for the higher injected activity of ^177^Lu-rituximab the toxicity of the treatment had a strong impact on the median survival. The optimal injected activity for ^177^Lu-rituximab might lie in between 300 and 530 MBq/kg, where an optimal amount of long term responders could be found without life-threatening toxicity.

#### Body weight

**Fig 3:**
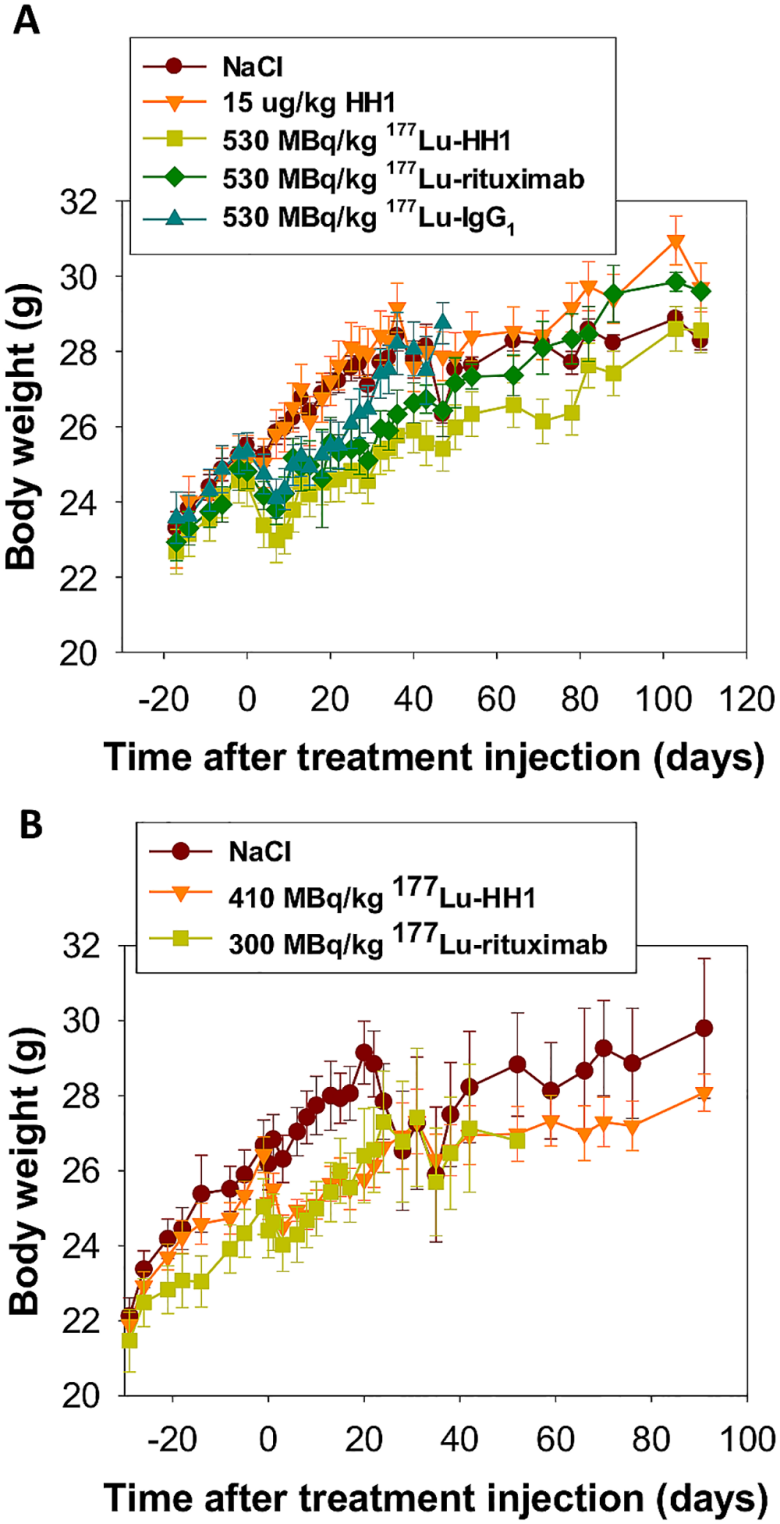
Average Body weight. Average body weight of nude mice with Ramos xenografts treated with 530 MBq/kg ^177^Lu-HH1, 530 MBq/kg ^177^Lu-rituximab, 530 MBq/kg ^177^Lu-IgG_1_ non-specific isotype control, 15 μg/kg HH1, NaCl 0.9%, 410 MBq/kg ^177^Lu-HH1 and 300 MBq/kg ^177^Lu-rituximab.

The average body weight of mice treated with RICs decreased sharply after injection. The average body weight loss lasted for around 7 days for treatment with 530 MBq/kg and around 4 days for treatment with 410 and 300 MBq/kg activities (Fig 3). Afterwards, the average body weight of the treated mice began to increase, tending toward the average body weight of the control groups.

#### Hematology

**Fig 4:**
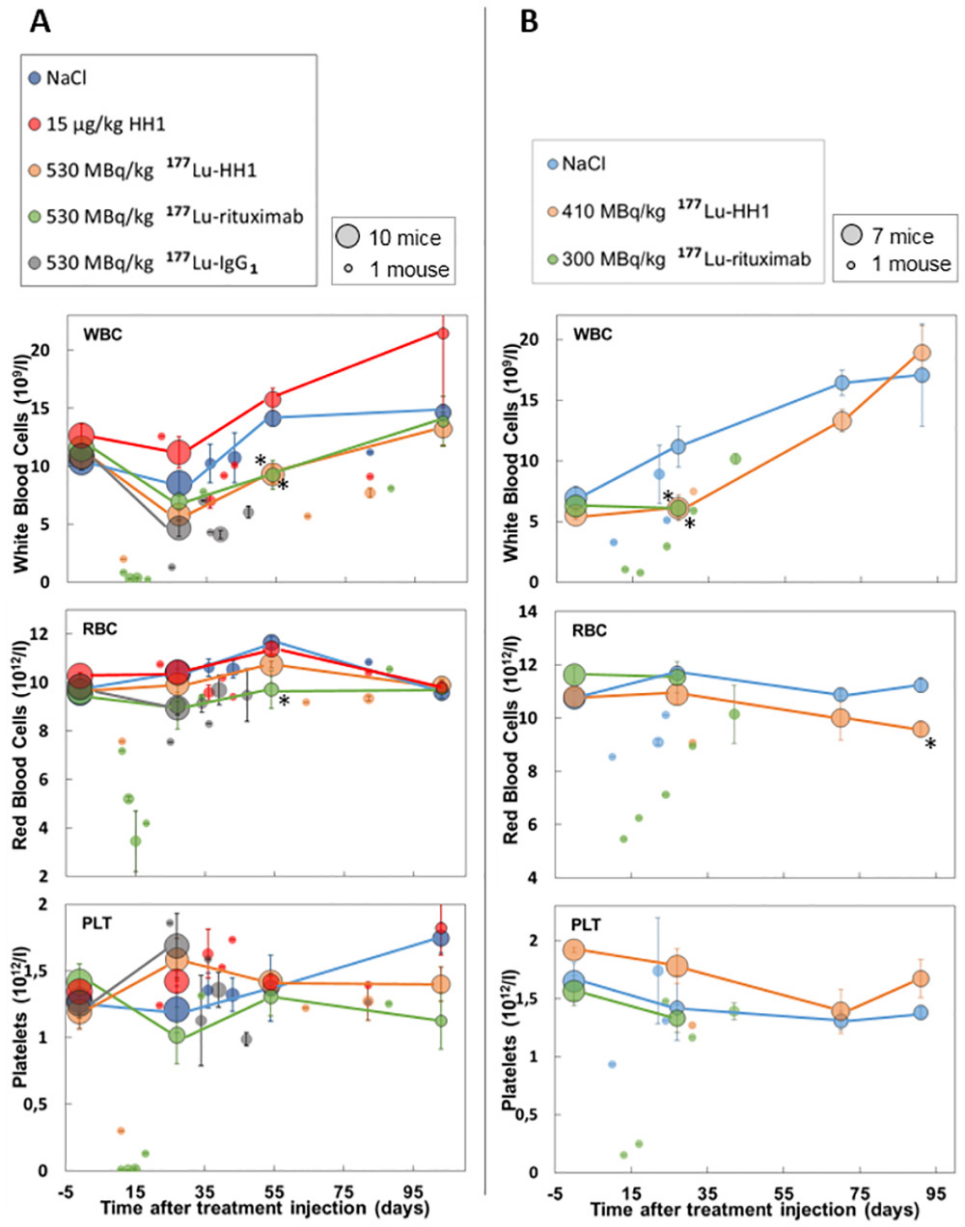
Hematology. Number of white blood cells (WBC), red blood cells (RBC) and platelets (PLT) in mice with Ramos xenografts treated with 530 MBq/kg ^177^Lu-HH1, 530 MBq/kg ^177^Lu-rituximab, 530 MBq/kg ^177^Lu-IgG_1_ non-specific isotype control, 15 μg/kg HH1, 0.9% NaCl (A) and 410 MBq/kg ^177^Lu-HH1 and 300 MBq/kg ^177^Lu-rituximab (B). The area of each circle represents the number of mice used to calculate the average values. Circles with dark rim and connected with lines represent samples taken at fixed time points during the study where all alive mice were sampled. Circles without dark rim and not connected by lines represent blood samples taken before euthanasia, where usually one or two mice were sacrificed. N = 1–10. Error bars = standard error. *: Significantly different from the corresponding 0.9% NaCl (control) group value (One-way ANOVA with Holm Sidak method for multiple comparisons, p < 0.05).

The average numbers of White Blood Cells (WBC) and Red Blood Cells (RBC) for mice treated with 530 MBq/kg of the different RICs were below the values for the NaCl control group for 4 and 8 weeks but normalized at the end of the study (Fig 4A). The average number of platelets (PLT) did not vary significantly between treatment and control groups. However, between 11 and 25 days after treatment with RICs, WBC, RBC and PLT numbers were considerably lower in mice treated with 530 MBq/kg, indicating a nadir around 2 weeks after treatment administration. There were no control animals sacrificed in this time range, but control values can be assumed to be close to those measured at baseline and around 4 weeks after treatment injection in the NaCl group.

The average number of WBC in mice treated with 410 MBq/kg ^177^Lu-HH1 and 300 MBq/kg ^177^Lu-rituximab was significantly lower than for the control group 4 weeks after treatment administration, but the values normalized towards the end of the experiment (Fig 4B). The average number of RBC and PLT were similar for all treatment groups (p > 0.05). There were no deaths associated to severe symptoms of radiation toxicity in this experiment. However, WBC counts in mice treated with ^177^Lu-rituximab that were sacrificed at 13 and 17 days due to tumor diameter higher than 20 mm had WBC, RBC and PLT values considerably lower than the values measured for NaCl at similar time points.

#### Histopathology

**Fig 5:**
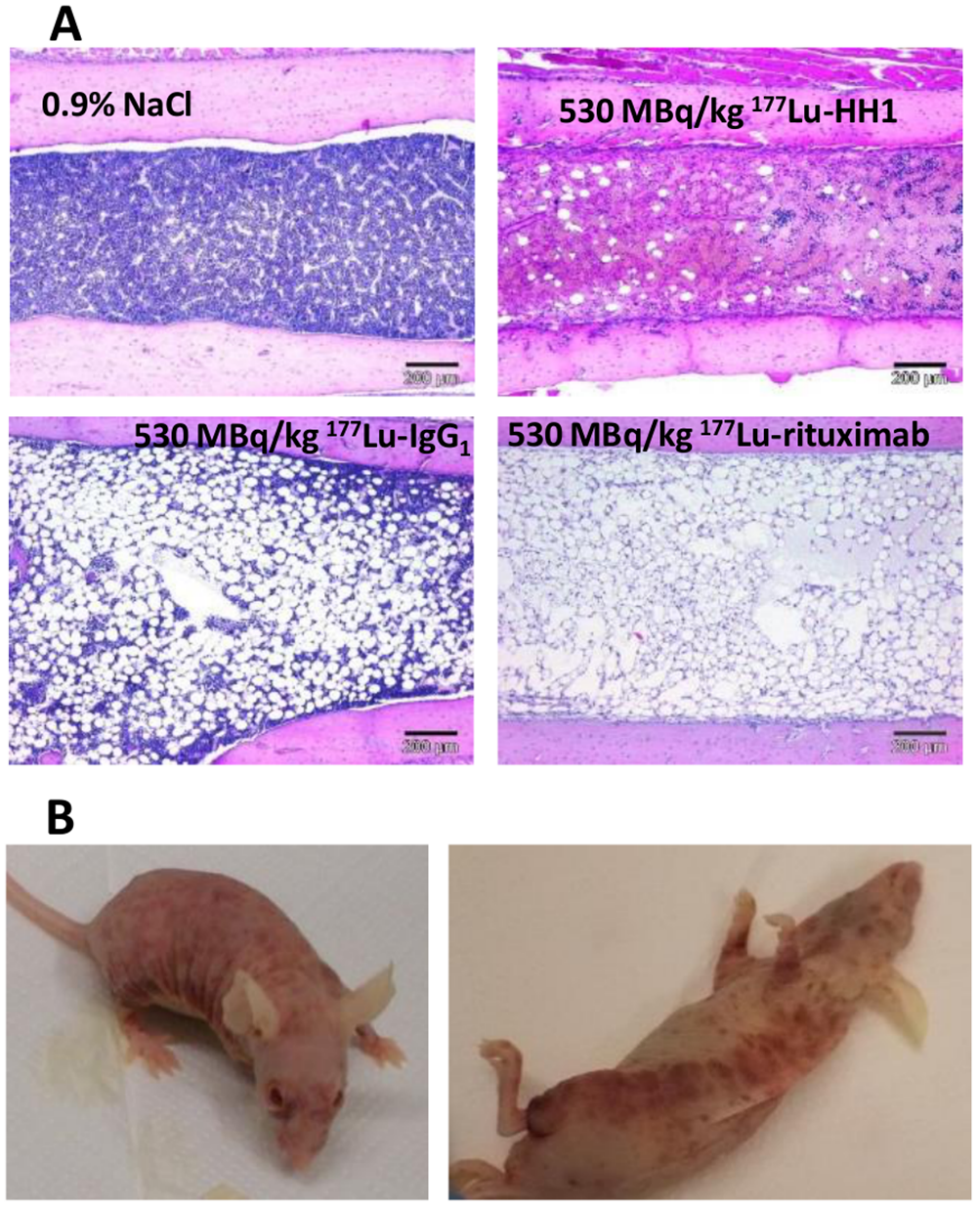
Histopathology. (A) Representative pictures of: a normal bone marrow from a mouse in the NaCl group (euthanized 109 days after treatment), marked reduced cellularity in a mouse treated with 530 MBq/kg ^177^Lu-HH1 euthanized 11 days after treatment, moderate reduced cellularity in a mouse treated with 530 MBq/kg ^177^Lu-IgG_1_ euthanized 25 days after treatment and severe reduced cellularity in a mouse treated with 530 MBq/kg ^177^Lu-rituximab euthanized 15 days after treatment. (B) Example of a mouse with severe skin hemorrhages 11 days after administration with 530 MBq/kg ^177^Lu-rituximab. The mouse was sacrificed the day the pictures were taken.

The main organs affected by treatment with RICs with activities equal to or higher than 530 MBq/kg were the bone marrow, lymph nodes, spleen and ovaries. Animals treated with 530 MBq/kg ^177^Lu-rituximab showed severe changes in hemato-lymphopoietic tissues, including decreased extra-medullary hematopoiesis, lymphoid depletion in the spleen and atrophy of the bone marrow (Fig 5A), indicating radiation damage (Table 3). These observations were more severe the sooner the mice had to be sacrificed after treatment injection. Similar observations were made for treatments with 800 and 1000 MBq/kg of ^177^Lu-HH1 [26]. Most of the mice showing severe radiation toxicity symptoms also presented skin hemorrhages (Fig 5B) that started as a skin rash a few days before euthanasia. The histopathology observations were in good agreement with the hematology measurements. For activities of 530 MBq/kg and higher ovaries were affected [26]. Mice euthanized between 11 to 25 days after treatment administration did, however, not show abnormalities in the ovaries, indicating that these lesions take more than 3 weeks to develop.

**Table 3:** Incidence of main histological findings in nude mice with Ramos xenografts treated with 530 MBq/kg ^177^Lu-HH1, ^177^Lu-rituxmab and ^177^Lu-IgG_1_ non-specific isotype control or 0.9% NaCl.

		Incidence (% (number of mice affected/total number of mice))			
Organ	Histological finding	NaCl	530 MBq/kg ^177^Lu-HH1	530 MBq/kg ^177^Lu-rituximab	530 MBq/kg ^177^Lu-IgG_1_
**Bone marrow**	Reduced cellularity	0 (0/10)	11 (1/9)	60 (6/10)	30 (3/10)
**Colon**	Lymphoid depletion	0 (0/9)	0 (0/9)	30 (3/10)	30 (3/10)
**Ileum**	Lymphoid depletion	0 (0/10)	11 (1/9)	10 (1/10)	10 (1/10)
**Liver**	Extramedullary hematopoiesis	0 (0/10)	0 (0/9)	0 (0/10)	40 (4/10)
	Inflamatory cell infiltration	50 (5/10)	11 (1/9)	10 (1/10)	0 (0/10)
**Ovaries**	Atretic follicles (present)	0 (0/10)	89 (8/9)	40 (4/10)	100 (8/8)
	Intersticial cell hyperplasia	0 (0/10)	78 (7/9)	40 (4/10)	50 (4/8)
**Spleen**	Extramedullary hematopoiesis	100 (10/10)	89 (8/9)	40 (4/10)	100 (10/10)
	Lymphoid depletion	10 (1/10)	22 (2/9)	60 (6/10)	80 (8/10)
	Pigmented macrophages	0 (0/10)	11 (1/9)	60 (6/10)	10 (1/10)

### Biodistribution and dosimetry

**Fig 6:**
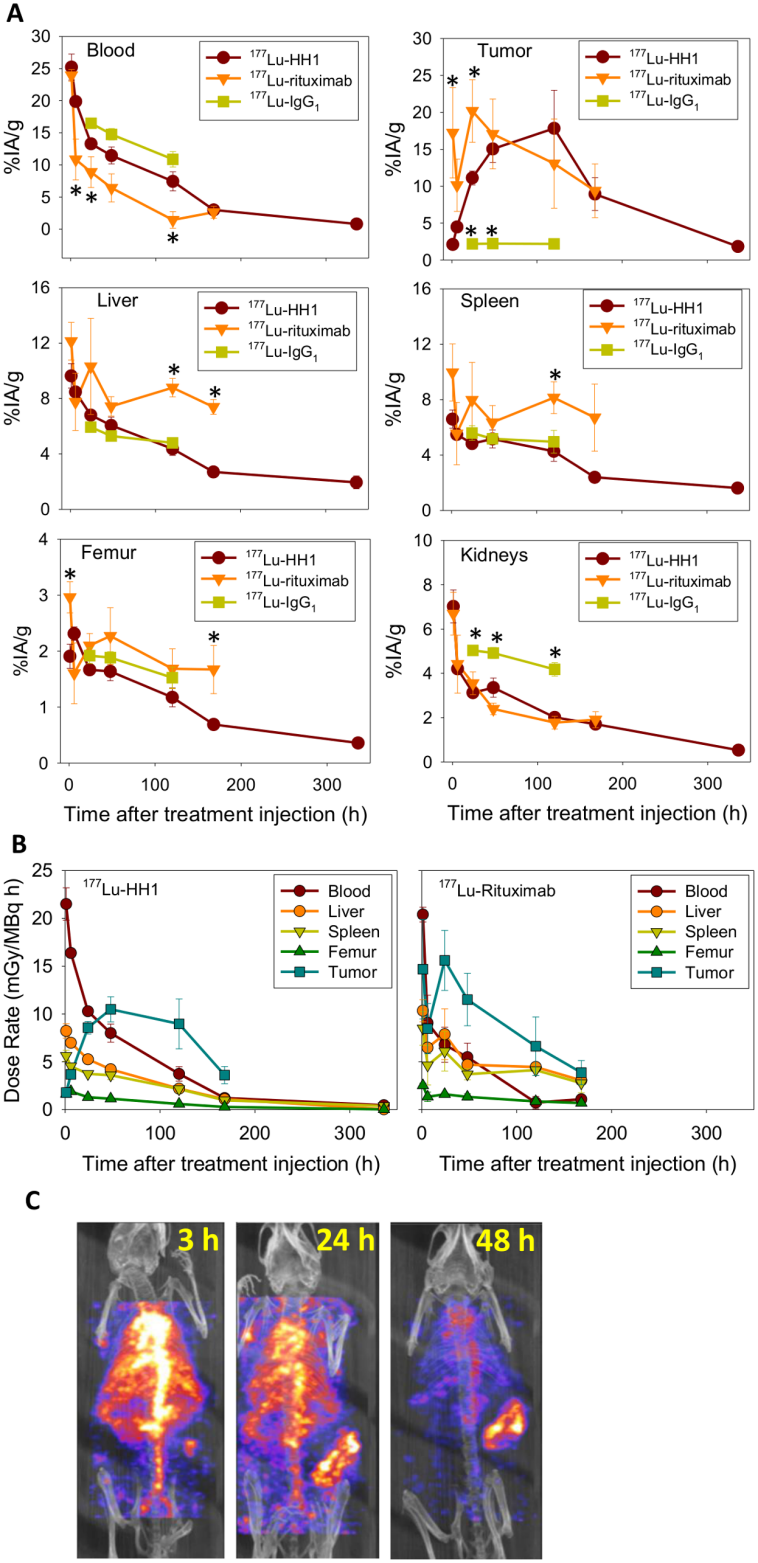
Biodistribution and dosimetry. (A) Uptake of ^177^Lu-HH1 (N = 6–8), ^177^Lu-rituximab (N = 4–5), ^177^Lu-IgG1 control (N = 5) in selected organs/tissues from nude mice with Ramos xenografts in percentage of injected activity per gram of tissue (%IA/g). Error bars = standard error. *: Significantly different from ^177^Lu-HH1 (One-way ANOVA with Holm Sidak method for multiple comparisons, p < 0.05). (B) Dose rate per MBq of injected activity in selected organs of nude mice with Ramos xenografts treated with ^177^Lu-HH1 (N = 6–8) and ^177^Lu-rituximab (N = 4–5). (C) *In vivo* SPECT/CT multiplanar rendered view of a nude mouse with a Ramos xenograft in its right flank injected with 10 MBq of ^177^Lu-HH1.

Blood clearance was slightly faster for ^177^Lu-rituximab than for ^177^Lu-HH1 (Fig 6A), which gave an absorbed dose to blood in mice treated with ^177^Lu-rituximab around half of that for ^177^Lu-HH1 (Table 4, Fig 6B). The retention of ^177^Lu-rituximab in liver, spleen and femur was significantly longer than that of ^177^Lu-HH1 or ^177^Lu-IgG_1_ (Fig 6A and 6B) and consequently absorbed doses to these organs were 2–3 times higher for treatment with ^177^Lu-rituximab than for ^177^Lu-HH1. The higher uptake of ^177^Lu-rituximab in femur (Table 4) probably resulted in a higher absorbed dose to red marrow, which is consistent with the higher toxicity and reduced bone marrow cellularity observed in mice treated with ^177^Lu-rituximab. The uptake in tumor was faster for ^177^Lu-rituximab, but the retention was shorter (not statistically significant, p > 0.05) than for ^177^Lu-HH1 (Fig 6A). The absorbed doses to tumor were close to 2 Gy for both ^177^Lu-HH1 and ^177^Lu-rituximab (Table 4). The uptake and retention of ^177^Lu-HH1 and ^177^Lu-IgG_1_ control were similar for all organs/tissues except for kidneys and tumor (Fig 6A).

**Table 4:** Absorbed radiation doses per injected MBq and tumor to normal tissues ratios in selected organs of nude mice with Ramos xenografts administered with ^177^Lu-HH1 and ^177^Lu-rituximab.

	Dose (Gy/MBq)		Tumor/Normal Tissue	
Organ/Tissue	^177^Lu-HH1	^177^Lu-rituximab	^177^Lu-HH1	^177^Lu-rituximab
Blood	1,26 ± 0,06	0,72 ± 0,1	1,7 ± 0,2	2,5 ± 0,5
Liver	0,71 ± 0,05	1,4 ± 0,1	2,9 ± 0,4	1,3 ± 0,2
Spleen	0,54 ± 0,01	1,3 ± 0,3	3,8 ± 0,5	1,3 ± 0,26
Kidney	0,36 ± 0,01	0,36 ± 0,03	5,8 ± 0,8	5,0 ±- 0,9
Femur	0,093 ± 0,001	0,15 ± 0,02	22,3 ±- 3,0	12,0 ±- 2,4
Tumor	2,1 ± 0,3	1,8 ± 0,3	——-	——-

SPECT/CT images showed a high and heterogeneous tumor uptake compared with other tissues 48 hours after injection of ^177^Lu-HH1 (Fig 6C). Uptake in lungs, heart and aorta was high 3 hours after injection and decreased with increasing time, being considerably reduced after 48 hours. Uptake in tumor was low 3 hours after ^177^Lu-HH1 administration but increased with time and was higher than in other tissues after 48 hours. The difference of tumor uptake measured by SPECT and gamma counter was below 10%, which shows good agreement between measurements performed *in vivo* with SPECT and *ex vivo* by gamma counting.

### Internalization experiments

**Fig 7:**
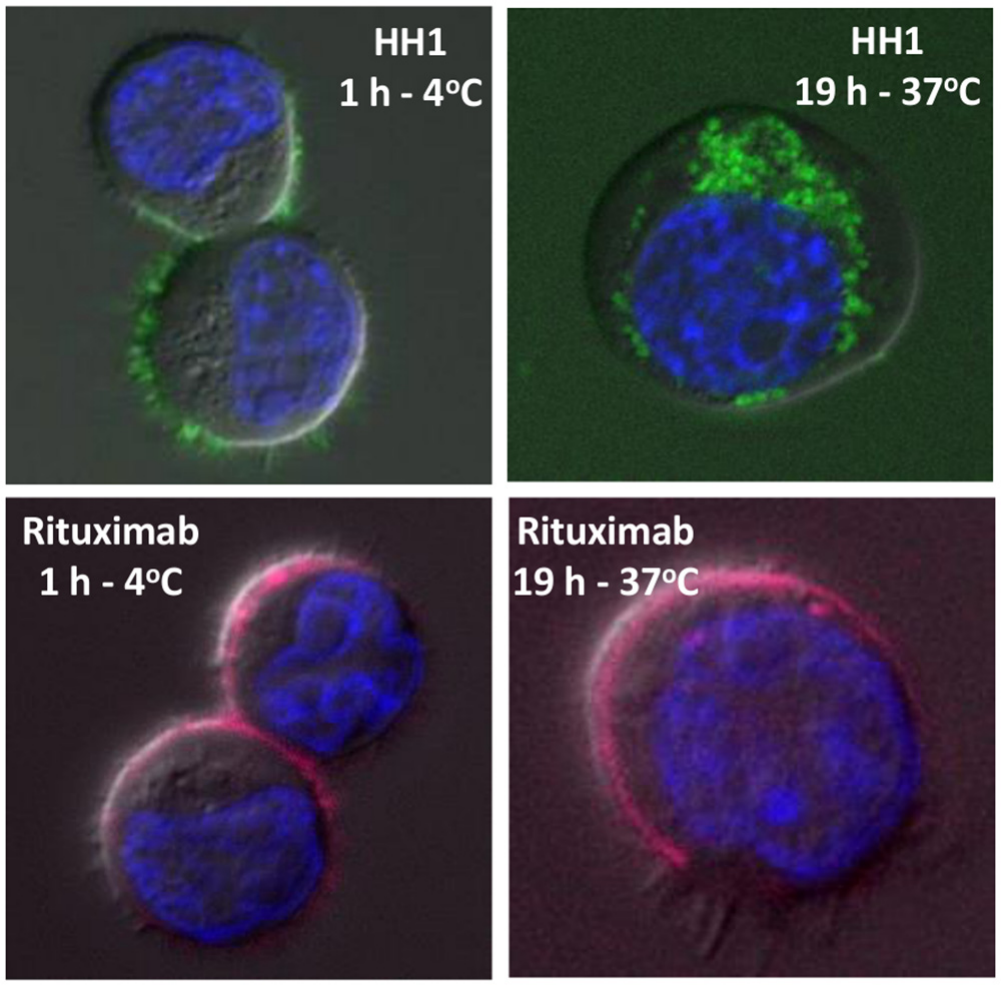
Internalization. Representative images of internalization of HH1 and Rituximab in Ramos cells after incubation with 10 μg/ml of HH1 or 20 μg/ml rituximab at 4°C or 37°C for 1 hour or 19 hours, respectively. HH1-DOTA bound to Alexa Fluor 488 is shown in green, Rituximab bound to Alexa Fluor 647 is shown in magenta and Hoechst 33342 bound to DNA in the cell nucleus is shown in blue. 20 to 40 cells were scanned for each treatment.

HH1 was almost completely internalized after 19 hours of incubation at 37°C, while there was no significant internalization after 1 hour incubation at 4°C. Some cells showed HH1 both on the cell surface and inside the cytoplasm while others only showed HH1 inside the cells after 19 hours at 37°C. A small degree of internalization of rituximab was observed after 19 hours of incubation at 37°C, while there was no sign of internalization after 1 hour incubation at 4°C. Fig 7 shows representative images of the cells.

### Binding experiments

The number of CD37 antigens per Ramos cell (B_max_) was half the number of CD20 antigens (Table 5). The dissociation constant (K_d_) of HH1 was lower than that of rituximab but the difference was not statistically significant.

**Table 5:** Maximum number of CD37 and CD20 antigens (B_max_) and equilibrium dissociation constant (K_d_) of ^177^Lu-HH1 and ^125^I-rituximab in Ramos cells.

Antibody (antigen)	K_d_ (nM)	B_max_ (antigens/cell)
**HH1 (CD37)**	1.6 ± 1	128000 ± 40000
**Rituximab (CD20)**	2.4 ± 1	279000 ± 51000

Results presented as average ± standard deviation. N = 2 for rituximab, N = 3 for HH1.

## Discussion

The majority of the NHL patients nowadays receive anti-CD20 mAbs as first line treatment. The first two RICs on the market, Zevalin and Bexxar, also targeted the same antigen. It has been shown that shaving or downregulation of the CD20 antigen might be a reason for resistance to rituximab [41–43] and it may also be the case for other CD20 based treatments. From a clinical perspective, it is plausible to use another target than CD20 for second line treatment, such as the CD37 antigen. There are currently four drug candidates, in addition to ^177^Lu-HH1, that target the CD37 antigen: an antibody-based protein [15], an Fc-engineered mAb[17] and two ADCs [19,20]. Given the heterogeneous vascularization and antigen expression normally found in tumors [44,45] it might be useful to develop a treatment that possess cross-fire contribution. We have therefore developed a new RIC (Betalutin) based on ^177^Lu linked to the anti-CD37 antibody HH1 [24]. The β-emissions of ^177^Lu have a mean range of 0.67 mm and are able to reach and kill even those cells to which the RIC is not able to bind. A series of pre-clinical studies have shown that ^177^Lu-HH1 successfully binds to both lymphoma cell lines and biopsies from NHL patients, indicating that targeting of the CD37 antigen with HH1 is clinically relevant [7]. Furthermore, the biodistribution of ^177^Lu-HH1 was found to be relevant for the treatment of NHL in SCID mice i.v. injected with Daudi lymphoma cells and in nude mice with Daudi [28] and Ramos subcutaneous xenografts as shown in Fig 6. In addition, *in vivo* experiments in SCID mice showed that ^177^Lu-HH1 had a strong therapeutic effect, increasing the survival of treated mice compared with mice treated with unlabeled HH1 and NaCl [7]. The current study shows that ^177^Lu-HH1 has strong antitumor activity and lower toxicity compared with ^177^Lu-rituximab in nude mice with subcutaneous Ramos xenografts. Even though Ramos cells had a higher expression of CD20 than CD37, the absorbed doses to tumor for ^177^Lu-HH1 and ^177^Lu-rituximab were similar, which might be related to the higher internalization of the CD37-HH1 complex than of the CD20-rituximab, as observed *in vitro*. It has been reported previously that human cells are highly heterogeneous in their ability to internalize CD20 when bound to mAbs [46], ranging from lack of internalization [8,47–49] to high internalization [50–53]. The higher myelotoxicity of ^177^Lu-rituximab than of ^177^Lu-HH1 might be explained by a higher uptake and retention of ^177^Lu-rituximab in the bone marrow as indicated by the higher uptake and retention in the femur, spleen and liver (Fig 6). The higher uptake and retention of ^177^Lu-rituximab in femur and spleen was not correlated with the high uptake and retention in the blood, but for myelotoxicity the cumulated activity in the bone marrow is more relevant than the cumulated activity in the blood. The uptake and retention in bone marrow is probably estimated better by the biodistribution data for femur than for blood. Another mechanism that might have played a role for the difference in toxicity and therapeutic effect of ^177^Lu-rituximab and ^177^Lu-HH1 is the difference in shaving of the antigen-antibody complex for CD20:rituximab and CD37:HH1 [52,54]. Treatment with ^177^Lu-HH1 had a strong anti-tumor effect in the nude mice even though the tumor uptake was inhomogeneous as shown in the SPECT/CT images. This result implies that cross-fire effect might play an important role in the therapeutic effect of ^177^Lu-HH1.

The toxicity data presented indicates that the dose-limiting organ is the bone marrow, which is in agreement with previous studies in mice and humans showing that the most common and dose-limiting side effect of RIT is bone marrow toxicity [26,55]. The maximum tolerated activities (MTA) in nude mice can be assumed to be 550 MBq/kg = 1667 MBq/m^2^ for ^177^Lu-HH1 and 400 MBq/kg = 1212 MBq/m^2^ for ^177^Lu-rituximab (allometric equivalence performed on the basis of Body Surface Area [56–60]). These values are in relatively good agreement with the clinical MTA of ^177^Lu-rituximab found by Forrer *et al*. of 1665 MBq/m^2^ in humans [61].

CD37 internalizes, and has modest shedding in transformed B-cells expressing this antigen [8,47]. The early studies with ^131^I-MB-1 have probably underestimated the potential of CD37 as target for RIT due to the use of a “non-residualizing” radiolabel. We have previously shown that the use of the “residualizing” radiolabel ^177^Lu chelated via a DOTA linker to HH1 increased the uptake of the RIC in tumor compared to the RIC ^125^I-HH1 up to 20 times [23]. This could at least partly be related to the accumulation of “residualizing” ^177^Lu inside the cells due to the internalizing nature of CD37 [23].

In summary, ^177^Lu-HH1 is a promising RIC as treatment against CD37-expressing NHL and the antitumor activity and the toxicity profile found in the preclinical studies supports further clinical investigation.

## Conclusion

This paper has shown, through comparisons of treatments of ^177^Lu-HH1, ^177^Lu-rituximab and non-specific ^177^Lu-IgG_1_ isotype control in nude mice with subcutaneous Ramos lymphoma xenografts, that ^177^Lu-HH1 has very promising properties as a treatment of NHL. ^177^Lu-HH1 considerably delayed tumor growth, increased the number of complete remissions and increased the survival of nude mice. In addition, ^177^Lu-HH1 was less toxic than ^177^Lu-rituximab. Based on these findings we conclude that ^177^Lu-HH1 is a promising agent for treatment of NHL and clinical evaluation is recommended.

## Supporting Information

S1 File Preliminary Experiments. Therapy results from the preliminary experiments that assisted in the design of the experiments presented in the paper.
(PDF)
