# Supplementary material for: Targeted Cancer Therapy with a Novel Anti-CD37 Beta-Particle Emitting Radioimmunoconjugate for Treatment of Non-Hodgkin Lymphoma
Source: PLoS One. 2015 Jun 11;10(6):e0128816. doi: 10.1371/journal.pone.0128816 (PMC4466226; doi:10.1371/journal.pone.0128816)
Supplement: S1 File — Therapy results from the preliminary experiments that assisted in the design of the experiments presented in the paper. (PDF) [file pone.0128816.s001.pdf]

S1 File. Preliminary Experiments and animal model.

Results from the preliminary therapy experiments that assisted in the design of the experiments presented in the main paper.

The materials and methods used were the same as described in the paper. Mice were implanted with Ramos xenografts in their right flank. Table S1 shows the details of each experiment. The results of the toxicity studies of these experiments have been presented previously[1]. There was a delay in tumor growth in mice treated with doses at or higher than 400 MBq/kg ^{177}Lu-HH1 (Figure S1 and S2) but there was no evident tumor growth delay in mice treated with 50 to 200 MBq/kg ^{177}Lu-HH1 when compared with NaCl. The tumor growth delay was more evident in mice treated with 800 and 1000 MBq/kg ^{177}Lu-HH1 but the toxicity associated with these dose levels was high: 50 % of the mice treated with 800 MBq/kg and 100 % of the mice treated with 1000 MBq/kg had to be sacrificed between 15 and 24 days after treatment injection due to symptoms of severe radiation toxicity[1]. Tumor growth in control mice or mice treated with 15 μg/kg HH1 was non-homogeneous with some tumors spontaneously regressing. Median survival time was between 18 and 24 days for mice treated with 400 MBq/kg ^{177}Lu-HH1 while it was between 6 and 17 days for the other treatment groups. The difference was not statistically significant ($p > 0.05$, Log-rank test), which might be related to the high amount of natural regressions in the control groups.

The dose of ^{177}Lu-HH1 had to be between 400 and 800 MBq/kg to give a therapeutic effect with acceptable toxicity. Therefore, a new experiment with a dose of 530 MBq/kg was performed (experiment 1 in paper).

Table S1. Overview of therapy and toxicity experiments in nude mice with Ramos xenografts.

Experiment	Treatments	mice/group	Mice age[a] (weeks)	Mice weights[b] (g)	Tumor volumes[b] (mm^3)
**1**	50 MBq/kg ^{177}Lu-HH1<br>100 MBq/kg ^{177}Lu-HH1<br>200 MBq/kg ^{177}Lu-HH1<br>400 MBq/kg ^{177}Lu-HH1<br>0.9% NaCl	4-5	7-10	21.9 - 28.8 (25 ± 2)	141 - 584 (320 ± 127)
**2**	400 MBq/kg ^{177}Lu-HH1<br>800 MBq/kg ^{177}Lu-HH1<br>1000 MBq/kg ^{177}Lu-HH1<br>15 µg/kg HH1<br>0.9% NaCl	9-10	8-11	18.5 - 24,5 (22 ± 2)	32 - 460 (140 ± 70)

[a]at implantation of xenografts

[b]at treatment injection Min-Max (Average ± SD)

Figure S1. Individual tumor growth after treatment with 50, 100, 200 and 400 MBq/kg ^{177}Lu-HH1 and 0.9 % NaCl.

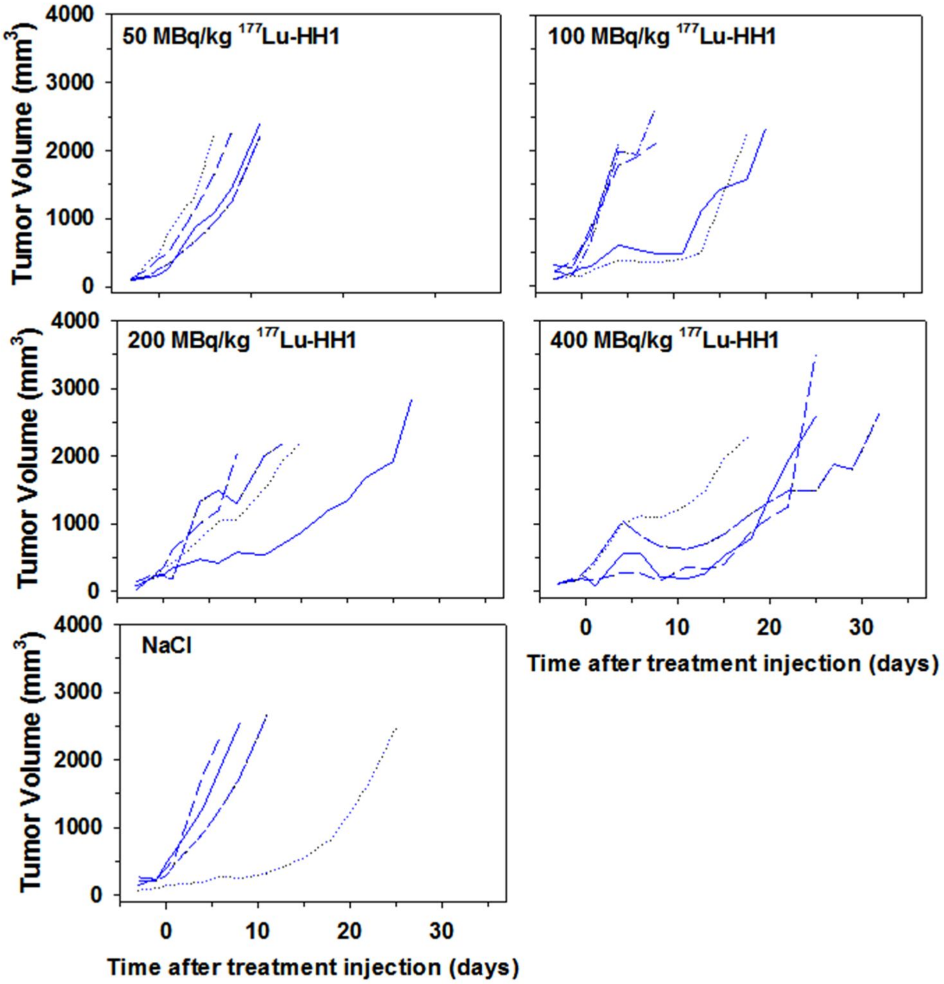

Figure S2. Individual tumor growth after treatment with 400, 800 and 1000 MBq/kg ^{177}Lu-HH1, 15 µg/kg HH1 or 0.9 % NaCl (n = Number of tumors in remission at the end of study/total number of initial tumors).

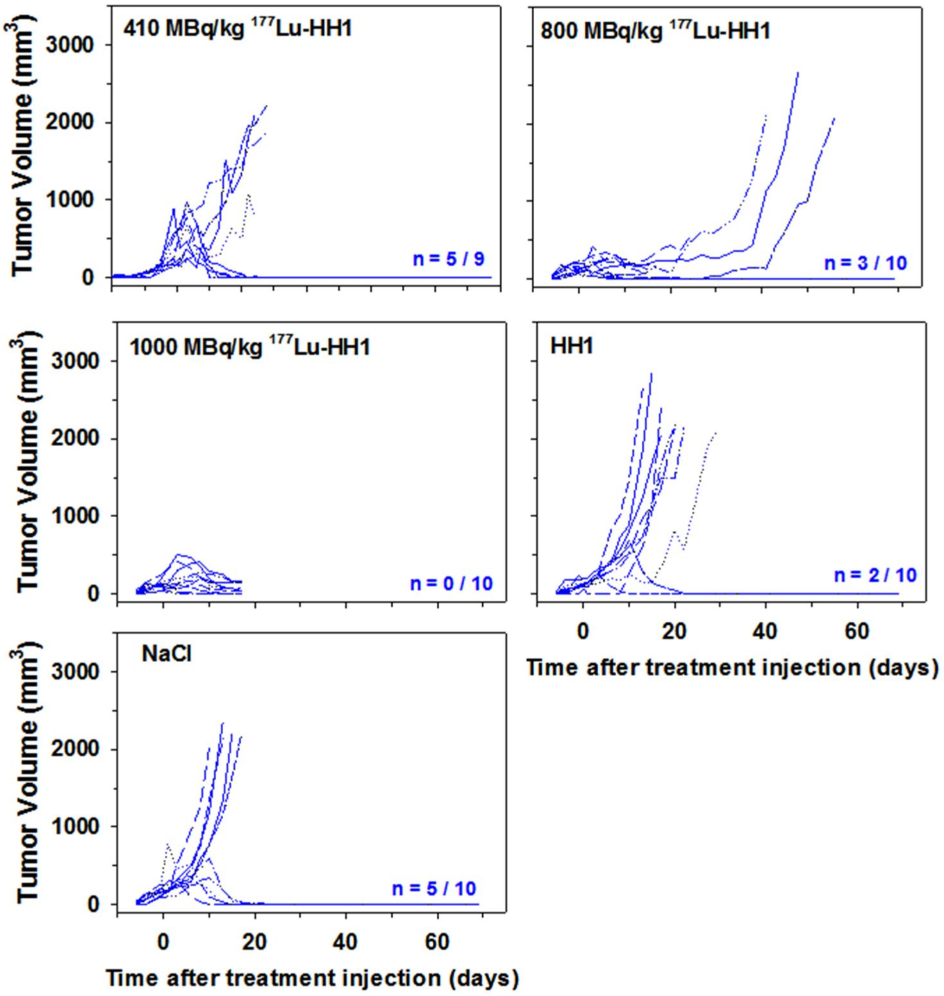

References

1. Repetto-Llamazares AH, Larsen RH, Giusti AM et al. 177Lu-DOTA-HH1, a Novel Anti-CD37 Radio-Immunoconjugate: A Study of Toxicity in Nude Mice. PLoS.One. 2014;9:e103070.
